# Has *Chlamydia trachomatis* prevalence in young women in England, Scotland and Wales changed? Evidence from national probability surveys

**DOI:** 10.1017/S0950268819000347

**Published:** 2019-03-04

**Authors:** D. Z. Kounali, N. J Welton, K. Soldan, S. C. Woodhall, J. Kevin Dunbar, S. J. Migchelsen, C. H. Mercer, P. Horner, A. E. Ades

**Affiliations:** 1 Population Health Sciences, Bristol Medical School, Oakfield House, Oakfield Grove, Bristol BS8 5BN, UK; 2 National Institute for Health Research Health Protection Research Unit in Evaluation of Interventions, University of Bristol, Bristol, UK; 3 Blood Safety, Hepatitis, Sexually Transmitted Infections and HIV Division, Public Health England, London, 61 Colindale Avenue, London NW9 5EQ, UK; 4 Institute for Global Health, University College London, Mortimer Market Centre, London, WC1E 6JB, UK

**Keywords:** *Chlamydia trachomatis*, prevalence, sexually transmitted infections (STIs)

## Abstract

We evaluate the utility of the National Surveys of Attitudes and Sexual Lifestyles (Natsal) undertaken in 2000 and 2010, before and after the introduction of the National Chlamydia Screening Programme, as an evidence source for estimating the change in prevalence of *Chlamydia trachomatis* (CT) in England, Scotland and Wales. Both the 2000 and 2010 surveys tested urine samples for CT by Nucleic Acid Amplification Tests (NAATs). We examined the sources of uncertainty in estimates of CT prevalence change, including sample size and adjustments for test sensitivity and specificity, survey non-response and informative non-response. In 2000, the unadjusted CT prevalence was 4.22% in women aged 18–24 years; in 2010, CT prevalence was 3.92%, a non-significant absolute difference of 0.30 percentage points (95% credible interval −2.8 to 2.0). In addition to uncertainty due to small sample size, estimates were sensitive to specificity, survey non-response or informative non-response, such that plausible changes in any one of these would be enough to either reverse or double any likely change in prevalence. Alternative ways of monitoring changes in CT incidence and prevalence over time are discussed.

## Introduction

*Chlamydia trachomatis* (CT) is the most commonly diagnosed sexually transmitted infection and a substantial cause of reproductive ill-health in women [1]. The National Chlamydia Screening Programme (NCSP) was introduced in England in 2003 and was active nationwide by 2008. The objectives of the NCSP have been twofold: to protect individual young women who are infected from reproductive damage by identifying and treating them and their partners, and to lower the population prevalence, and thereby lower the risk of CT transmission. The NCSP has been operational with targets designed to meet these objectives for over 10 years, although doubts about its benefits continue to be raised [2–6]. In 2017, over 1.3 million chlamydia tests were carried out among young people aged 15–24 years, of which 9.8% were positive. Assuming one test per person, an estimated 28% of young females and 11% of young males were tested for chlamydia [7].

The National Surveys of Sexual Attitudes and Lifestyles (Natsal) [8–10] are key sources of policy-relevant data on reproductive and sexual health in Great Britain. The Natsal-2 and -3 surveys undertaken in 2000–01 and 2010–12, respectively, were stratified cross-sectional probability sample surveys of the general population resident in England, Scotland and Wales. The surveys included inviting a random sub-sample of survey responders aged 16–44 years to provide urine samples which were tested for CT by Nucleic Acid Amplification Tests (NAATs). Previous analyses of Natsal data have suggested that CT prevalence in 18–24 years old women had barely changed between 2000, before the introduction of the NCSP (3.1%; 95% CI 1.8–5.2 in 18–24 years old), and 2010 after its introduction (3.2%; 95% CI 2.2–4.6) [8]. These figures include adjustments for the different distributions of prevalence-related covariates in responders and non-responders. Subsequently, adjustments for assay sensitivity and specificity were explored [11].

The objective of this paper is to evaluate the utility of Natsal surveys as a data source on CT prevalence and CT prevalence change between 2000 and 2010, during which time screening was introduced. More generally, we want to highlight the methodological issues that arise when using surveys of this kind to estimate the population prevalence of sexually transmitted infections. The original rationale for this work was to support a separate exercise of modelling age- and time-specific seroprevalence data from surveys such as the PHE Seroepidemiology Unit [12] and Health Survey for England [13] to recover estimates of age- and time-specific incidence. Both surveys would generate predictions of age-specific incidence in 2000 and 2010, and the intention had been to validate results against estimates of CT incidence, which would be derived from Natsal CT prevalence using estimates of the duration of CT infections from Price *et al*. [14]. Ultimately, it was hoped that the Natsal estimates could be used to calibrate the estimates from seroprevalence models using multi-parameter evidence synthesis [15].

Several issues require consideration before drawing conclusions about CT prevalence from these surveys. First, the overall net response rates, defined as the proportion of those invited to participate in the original Natsal surveys, who were invited to provide a urine sample, and who did so, were very low: 45.4% and 34.6% in 2000 and 2010, respectively [9]. The non-participation bias corrections that have been published [8, 11, 16] consist of inverse propensity score (PS) reweighting [17], which reweights the responding population so that its covariate distribution matches that of the target population. The effect of these adjustments was to *lower* CT prevalence estimates [10], apparently because groups at higher risk of CT infection (e.g. those reporting a higher number of partners, heterosexual anal sex and STI diagnosis) were more prevalent among responders than non-responders. The premise underlying PS reweighting is that, at any specific covariate pattern, the prevalence in responders and non-responders is identical, so that the only adjustment that is required is to reweight the covariate distribution in the responders so that it matches the overall (target) population.

This premise is not supported by the ClaSS study [18, 19], where CT prevalence among those who responded to the initial invitation was considerably lower than in those who responded to the subsequent invitations, although this is not adjusted for covariates. An adjustment for non-response that addresses this will *increase* the estimate of CT prevalence [11].

A further complication is the Natsal-2 and -3 surveys used different NAATs to measure CT prevalence. The Abbot Diagnostics Ligase chain reaction (LCx) test [8] was used in Natsal-2, and the Aptima AC2 followed by a further confirmatory assay in Natsal-3. Woodhall (2015) [11], using data from a study comparing the two assays [20], concluded that the sensitivity of LCx relative to AC2 was 91.1% (i.e. using AC2 as a gold standard), and its specificity was 99.1% (95% credible interval (CrI) 98.0–99.7). However, it can be shown (see Appendix 1) that such a low specificity is not compatible with a CT prevalence as low as the 0.75% that was observed in 35–44 years old women, as it implies, we should expect a 0.9% prevalence *due to false positives alone*.

In this paper, we consider a range of adjustments: for test accuracy, for propensity to respond to the urine survey and for non-response bias, to make some quantitative projections regarding changes in CT prevalence between 2000 and 2010, based on the Natsal surveys. Our intention is not so much to generate definitive estimates; it is rather to generate a range of estimates based on reasonable assumptions, in order to identify the key drivers of uncertainty, in the hope of guiding future research efforts. We include both statistical uncertainties originating in sampling variation, and structural uncertainty which arises from uncertain modelling assumptions.

## Methods

We adopted a Bayesian approach, using Markov chain Monte Carlo in WinBUGS 1.4.3 [21]. Throughout convergence was achieved within 10 000 iterations. Posterior inference was based on a total 200 000 samples from two chains after discarding the first 20 000.

Our target parameters are the age-specific CT prevalence in women in 2000 and 2010 in the general population resident in England, Scotland and Wales, with age grouped into the ranges 16–17, 18–19, 20–24, 25–29, 30–34 and 35–44. From these we derived estimates of CT prevalence change between 2000 and 2010. The process of mapping from the CT prevalence observed in the Natsal surveys to unbiased estimates of CT prevalence in the general population can be conceptualised in four steps:

1. Adjustments for sensitivity and specificity of the assays.
2. Adjustment for restriction of the urine survey to the sexually experienced population.
3. EITHER (a) Use of PSs to re-weight the numerators and denominators in the urine sample population so that the covariate distribution matches the general population.
4. OR (b) Further adjustments for non-response to allow for differences in CT prevalence in responders and non-responders.

We now detail each step.

### Crude unadjusted estimate

The observed data are the binomial numerator *r*_*a*_ and denominator *n*_*a*_ at each age *a*. We begin with the crude, unadjusted, estimate of prevalence 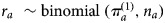, using a vague prior 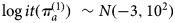 centred on a prevalence of approximately 0.5% (95% CrI 1E-10–2.5E7). This ensures that prior has negligible impact on the posterior results.

### Adjustment for sensitivity and specificity in NAAT assays

Using a copy of the data, and the same prior (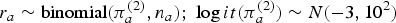), we estimate a second parameter 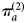. Then, an estimate of CT prevalence adjusted for sensitivity and specificity, 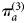, can be recovered via the relationship:
1
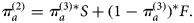


Note that the estimates 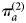 are updated by both the observed data *and* the sensitivity and specificity corrections, and their posteriors therefore differ from 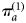. 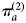 thus represents the prevalence we would expect to observe if there was no correction for sensitivity and specificity, while accounting for imperfect test accuracy.

We have assumed that the joint specificity of AC2 and its confirmatory test is 99.9% (95% CrI 99.7–99.99). Regarding the specificity of LCx, a series of analyses in the 1990s [22–24] suggested that the specificity of LCx was around 99.9%. These methods employed what was referred to as an ‘expanded reference standard’. Subsequently, this method, under the name of ‘discrepancy analysis’, was subjected to repeated criticism from statisticians [25], to the extent that it is no longer used. Nevertheless, other investigators using a methodology based on repeat testing confirmed that the specificity of LCx was likely to exceed 99.5% [26]. Accordingly, we have assumed that the specificity of LCx was 99.8%, with a 95% CrI approximately 99.7–99.9%. Sensitivity analyses were run assuming specificity of 99.5% with 95% CrI 99.1–99.9%.

For sensitivity of AC2, we carried out a random-effects meta-analysis of five studies and obtained a mean estimate 94.95% with 95% CrI 93.39–96.52 (see Appendix 2 for details). For LCx we have set LCx per cent sensitivity at 1.5 points below AC2, i.e. at 93.45%, and have run a sensitivity analyses in which sensitivity is 3.0% points below AC2 (91.95%), approximately the same as estimates based on Gaydos [20].

These assumptions about sensitivity and specificity of LCx differ somewhat from those that have been used in previous analyses [11], which were based on Gaydos [20]. Appendix 1 presents an analysis which suggests that the specificity assumed (99.1%) was unrealistically low and incompatible with the observed Natsal-2 data.

We assumed throughout that prevalence would be allowed to influence the test accuracy parameters.

### Estimates for the general population

The Natsal-2 urine survey and the Natsal-3 urine survey were designed to estimate CT prevalence in the sexually experienced population aged 18–44 years. If the proportion who were sexually experienced in age group *a* is *κ*_*a*_ we adjust the estimates 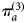 as follows: 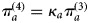.

### Construction of balancing weights

The probability *θ*_*i*,*a*_ that a woman *i* aged *a* provided a valid urine sample, given that she is a member of the general female population aged *a* was estimated by a logistic regression: 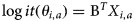 where 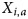 is a vector of covariates and 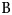 their estimated coefficients. From this we derive weights for each individual woman, 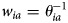 in the survey. We have used the same weights as in earlier studies [8, 11], slightly adjusted because eligibility for the urine surveys was those participants reporting that they were sexually experienced and our target parameter is CT prevalence in the general population aged 16–44. Among the key variables adjusted for were geographic region, educational status, the total number of sexual partners, sexual behaviour and previous STI diagnoses. Using the individual weights *w*_*ia*_ we can form weights applicable to the aggregated numerators and denominators:

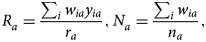
where *y*_*ia*_ is an indicator, 1 if *i*was CT+ and otherwise 0. We can derive an estimate of prevalence rebalanced to allow for the different covariate mix in responders and non-responders: 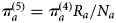. Note that these rebalanced estimates incorporate the corrections for sensitivity and specificity and sexual behaviour. Note also that the weights are assumed to be estimated without error.

### Adjustment for non-response

In the ClaSS study, CT prevalence in those responding to the subsequent postal invitation, phone call or home visit was higher than those responding to the initial postal invitations, with an odds ratio (OR) of 1.48 (95% CrI 1.03–2.11). We construct a sixth estimate of CT prevalence, which adjusts the population estimates 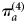, as follows:

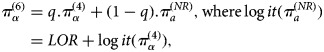
where *LOR* is the log OR for CT prevalence in non-responders relative to responders, and *q* is the probability of responding. Note that while the adjustments for non-response and PS reweighting are both attempts to address biases arising from the incomplete response to the survey and the likelihood that non-responders have different CT prevalence, we view them as *alternatives*, and therefore do not apply both. This is taken up further in discussion.

In the ClaSS study, the response rate to the initial postal invitations in women aged 16–24 years was only 25.3%, going up to 30.4% after a second postal invitation, which is almost identical to the net response rate to the urine sample in Natsal-3, and eventually to 36.4% at a third follow-up. In our base-case analysis, we apply the OR from the ClaSS study (1.48) to the Natsal-2 and Natsal-3 surveys, conservatively assuming that the same OR applies to all non-responders in both surveys. As a sensitivity analysis, we consider a higher OR (1.90), which is well within the 95% CrI of the ClaSS estimate (95% CrI 1.0–2.1). We also examine scenarios in which the OR is greater by 10% in Natsal-3, on the basis that response rate was lower and so the CT prevalence in non-responders likely to be relatively higher. This gives adjustments as follows: Natsal-2: 1.48, Natsal-3: 1.63; and Natsal-2: 1.90, Natsal-3: 2.09.

The purpose of this paper is to reveal the nature and the extent of the uncertainties in estimates of prevalence change. We therefore document the span of the posterior credible intervals arising from sampling uncertainty in the original data and in the other inputs, and the impact of the sensitivity analyses on the posterior mean change in prevalence. The WinBUGS code and datasets are given in full in Appendix 3, so that readers can examine other outputs and carry out further sensitivity analyses.

## Results

Table 1 sets out age-specific CT prevalence estimates from Natsal-2 and -3, respectively, starting with the crude estimates, then sensitivity-specificity-adjusted estimates. The latter adjustments are incorporated into both the PS reweighted estimates, and the response-bias corrected estimates.

**Table 1.** 

			Crude prevalence 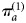		Sens/Spec corrected^a^ 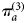			PS re-weighting 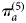		Non-response corrected^b^ 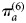	
Age group	CT+	*N*	Mean	95% CrI^c^	Mean	95% CrI		Mean	95% CrI	Mean	95% CrI
(a) Posterior mean CT prevalence and change in prevalence in 2000 in women according to age, without adjustment (crude prevalence) and under different adjustments for sensitivity and specificity, propensity score reweighting for covariate imbalance and correction for informative non-response^d^.											
Natsal-2: 2000											
18–19	4	103	3.89	(1.1–8.3)	3.83		(0.7–8.6)	3.45	(0.7–7.7)	4.66	(0.9–10.6)
20–24	12	276	4.35	(2.3–7.0)	4.42		(2.2–7.4)	2.67	(1.3–4.4)	5.50	(2.6–9.4)
25–29	11	400	2.75	(1.4–4.6)	2.70		(1.2–4.6)	2.05	(0.9–3.5)	3.41	(1.5–6.1)
30–34	9	472	1.91	(0.9–3.3)	1.78		(0.7–3.3)	1.35	(0.5–2.5)	2.28	(0.8–4.4)
35–44	6	804	0.75	(0.3–1.4)	0.41		(0.0–1.2)	0.33	(0.0–1.0)	0.52	(0.0–1.6)
Average 18–24			4.22	(2.4–6.5)	4.25	(2.3–6.7)		2.89	(1.5–4.6)	5.26	(2.8–8.6)
Average 25–44			1.54	(1.0–2.2)	1.32	(0.7–2.0)		1.02	(0.6–1.6)	1.69	(0.9–2.7)
(b) Posterior mean CT prevalence and change in prevalence in 2010 in women according to age, without adjustment (crude prevalence) and under different adjustments for sensitivity and specificity, propensity score reweighting for covariate imbalance and correction for informative non-response.											
Natsal-3: 2010											
16–17	5	171	2.93	(1.0–5.9)	2.91	(0.8–6.1)		2.32	(0.7–4.9)	3.82	(1.1–8.2)
18–19	11	224	4.91	(2.5–8.1)	5.03	(2.5–8.4)		3.88	(1.9–6.5)	5.33	(2.5–9.2)
20–24	21	597	3.52	(2.2–5.1)	3.57	(2.2–5.3)		2.55	(1.5–3.8)	4.36	(2.5–6.8)
25–29	16	650	2.46	(1.4–3.8)	2.45	(1.3–3.8)		2.16	(1.2–3.4)	3.16	(1.6–5.3)
30–34	7	496	1.41	(0.6–2.6)	1.33	(0.4–2.6)		0.79	(0.2–1.5)	1.74	(0.5 –3.6)
35–44	2	527	0.38	(0.0–1.1)	0.14	(0.0–0.8)		0.10	(0.0–0.6)	0.18	(0.0–1.1)
Average 18–24			3.92	(2.7–5.3)	3.99	(2.7–5.5)		2.93	(2.0–4.1)	4.64	(2.9–6.9)
Average 25–44			1.16	(0.7–1.7)	1.01	(0.6–1.6)		0.79	(0.5–1.2)	1.32	(0.7–2.2)
Change 2000–2010											
Age 18–24			−0.30	(−2.8 to 2.0)	−0.27	(−3.0 to 2.2)		0.04	(−1.9 to 1.8)	−0.63	(−4.0 to 2.4)
Age 25–44			−0.38	(−1.1 to 0.4)	−0.31	(−1.2 to 0.5)		−0.23	(−0.9 to 0.4)	−0.37	(−1.5 to 0.7)
											

a LCx specificity 99.8%; AC2 specificity 99.9%; AC2 sensitivity 94.95%; LCx sensitivity 1.5% less than AC2.

c 95% Credible intervals.

b Non-response OR = 1.48 and remains the same for both 2000 and 2010.

d Each new adjustment is additional to sensitivity and specificity adjustments, e.g. propensity score re-weighting is applied on estimates corrected for imperfect sensitivity and specificity and adjustment for informative non-response is applied on estimates corrected for imperfect sensitivity and specificity.

The crude estimates of change suggest an absolute fall of 0.30 percentage points (95% CrI −2.8 to 2.0) in CT prevalence in 18–24 years old women from 4.22% to 3.92%. The span of the 95% interval is 4.8 percentage points, not only far exceeding the central estimate, but greater than the estimates of prevalence themselves. In 25–44 years old, there was a fall of 0.38 percentage points (95% CrI −1.1 to 0.4) from 1.54% to 1.16%, a credible span of 1.4 percentage points, again comparable with the incidence estimates themselves.

The effect of the sensitivity adjustment described in equation 1 is to increase estimated prevalence by a multiplicative factor, so the increase in prevalence is highest in younger women, who have the highest observed prevalence. However, the effect of the specificity adjustment reduces the estimates, as it interprets a proportion of the observed positives as false positives. This latter effect is more marked in the older groups, where observed prevalence is low (Table 1). Goodness of fit statistics (not shown) do not suggest a conflict between the prevalence data and the sensitivity and specificity assumptions. Thus, as seen in Table 1, the two adjustments tend to cancel out, so that following sensitivity and specificity corrections, the CT prevalence appears to have fallen by 0.27 percentage points in 18–24 years old with a slightly larger uncertainty span of 5.2 percentage points due to uncertainty in the adjustments, and by 0.31 percentage points in 25–44 years old with an uncertainty span of 1.7 percentage points (Table 1).

The net effect of the PS reweighting is to lower CT prevalence in almost every cell (Table 1), because responders tended to be disproportionately in groups with higher CT risk, as noted in earlier work [8]. The size of the correction was especially marked in 20–24 years old in Natsal-2 and 30–34 years old in Natsal-3. The net effect across all ages is similar in both surveys, with the predicted change in CT prevalence now being a rise of 0.04 percentage points in the younger group, and a fall of 0.23 percentage points in the older. The uncertainty intervals are shrunk downwards (3.7 and 1.3 percentage points in the younger and older, respectively) in approximately the same proportion as the prevalence estimates themselves. Note that the PS-reweighting methods used here [8] take no account of the uncertainty in the weights, so that posterior uncertainty is under-estimated.

If instead of PS reweighting, we apply the same non-response correction to both Natsal-2 and -3 (Table 1), based on the ClaSS data, then we estimate a fall of 0.63 percentage points between the two surveys for the 18–24 group, and a fall of 0.37 percentage points in the 25–44 group. However, due to the uncertainty in the ClaSS estimates, the uncertainty spans increase to 6.4 and 2.2 percentage points in the 18–24 and 25–44 years old groups, respectively.

Results of additional sensitivity analyses are given in Table 2. As with Table 1, three sets of results are given for each scenario: adjustment for sensitivity and specificity; the same adjustment with additional PS-reweighting; and the sensitivity and specificity adjustment with an adjustment for differential non-response. The first row (Scenario 0) is the base-case analysis from Table 1. In Scenario 1, all parameters are held at the base-case values except that the specificity of the LCx assay reduced from 99.8% to 99.5%. This very small change leads to a predicted increase in the prevalence of 0.49 in the younger group and 0.24 percentage points in the older, with similar results if either PS-reweighting or non-response adjustment is adopted as well. By contrast, results are robust against changes in NAAT sensitivity (Scenario 2 of Table 2). By attributing more of the observed Natsal-2 prevalence to false positives, the effect is to make it appear more likely that prevalence has risen. Perhaps surprisingly, estimates of change in prevalence are quite insensitive to an increase in the OR from 1.48 to 1.90 (Scenario 3). However, if we allow the OR correction to be 10% greater in Natsal-3 than in Natsal-2, which could be justified by the lower response rate in Natsal-3, this again acts to make prevalence appear to have risen, or to have fallen less (Scenarios 4 and 5).

**Table 2.** Posterior estimates for the mean change in CT prevalence between 2000 and 2010 under different scenarios

		Se-Sp-corrected		PS re-weighting		Non-response	
Scenario number	Sensitivity analyses scenarios	18–24	25–44	18–24	25–44	18–24	25–44
0	Base case^a^	−0.27	−0.31	0.04	−0.23	−0.63	−0.37
1	Prior specificity LCx = 99.5 (95% CrI 99.1–99.9)	0.49	0.24	0.60	0.20	0.29	0.33
2	Sensitivity LCx 3.0% less than AC2	−0.33	−0.33	−0.02	−0.24	−0.70	−0.39
3	OR 2000 = OR 2010 = 1.90	−0.27	−0.31	0.04	−0.23	−0.63	−0.41
4	OR 2000 = 1.48, OR 2010 10% higher	−0.27	−0.31	0.04	−0.23	−0.31	−0.28
5	OR 2000 = 1.90, OR 2010 10% higher	−0.27	−0.31	0.04	−0.23	−0.23	−0.29

a Base-case assumptions: prior LCx specificity 99.8 (95% CrI 99.7–99.9); LCx sensitivity 1.5% less than AC2.

Non-response OR = 1.48 and remains the same for both 2000 and 2010.

Table 3 summarises the impact of modelling assumptions examined in Table 2, by asking: ‘how much difference would it make to the estimate of CT prevalence change if we choose one modelling approach compared to another?’. The table shows how each modelling contrast is constructed from Table 2 results. Three modelling choices are examined. First (Row 1), adopting PS-reweighting instead of adjustment for informative non-response would change the estimate of prevalence change by 0.66% points in 18–24 years old, and 0.11 in 25–44 years old. Second, the choice between the 99.8% and 99.5% LCx specificity has an impact on CT prevalence change that is equal to, or larger than, the base-case prevalence changes in both age groups, however non-participation is handled (Row 2). Finally (Row 3), differential non-response adjustments in the 2000 and 2010 surveys have a major impact on predictions in the younger group, but less for the older.

**Table 3.** Impact summary of structural uncertainty: absolute difference (in bold) between different contrasting scenarios in posterior estimates of prevalence change, and how these are derived from Table 2, (in parentheses)

Contrast – difference in estimates	Age 18–24 difference in estimates of prevalence change	Age 25–44 difference in estimates of prevalence change
PS reweighting *vs*. non-response adjustment, base-case scenario	**(0.04 to −0.63):0.66**	(**−0.23 to −0.37):0.14**
99.5% LCx specificity *vs*. 99.8%		
(a) PS reweighting	**(0.60–0.04):0.56**	**(0.20 to −0.23):0.43**
(b) Non-response correction	**(0.29 to −0.63):0.92**	**(0.33to −0.37):0.70**
Non-response correction 2010 10% higher than 2000 *vs*. same OR = 1.48	(**−0.31 to −0.63):0.32**	(**−0.28 to −0.37):0.10**
Non-response correction 2010 10% higher than 2000 *vs*. same OR = 1.90	(−0.23 to −0.63):**0.40**	(−0.29 to −0.37):**0.09**

## Discussion

This paper provides a re-analysis of the CT prevalence estimates in Natsal-2 and -3, adding to the previous work of Sonnenberg *et al*. and Woodhall [8, 11], who applied PS re-weighting and sensitivity/specificity corrections. The analysis here differs from the earlier ones in several ways. First, we have altered the weights as our target was the general British female population aged 16–44, rather than the population of sexually experienced women. Second, we used slightly different corrections for sensitivity and specificity, and implemented them in a Bayesian way, which ensure that probability estimates remain in the (0.1) interval, and hence rule out negative prevalence estimates. Both test accuracy and PS reweighting corrections tend to slightly lower prevalence. Thirdly, as an alternative to PS re-weighting, we have assumed that non-responders have a different CT prevalence than responders, and we have used the OR for non-responder *vs.* responder from the ClaSS study [18, 19] to illustrate the potential impact of a non-response correction. This type of adjustment raises CT prevalence.

Our main finding is that the 95% CrI for observed CT prevalence change alone, setting aside the different methods and types of adjustment, itself spans 4.8%, obscuring even the largest prevalence change that could be considered plausible. This is the direct result of the small achieved sample size of the urine surveys.

A second major finding is that the impact of any one of three sources of structural uncertainty would be enough to either reverse or double any likely true change. We identified the following sources of structural uncertainty: method for correcting for non-response rate (PS reweighting or non-response correction), test specificity and different non-response corrections in the two surveys. Test sensitivity and overall level of non-response correction had little impact on results.

We conclude that although Natsal provides key data on sexual and reproductive health in the British general population, the surveys are unlikely to generate useful evidence on overall CT prevalence, or trends in CT prevalence, unless much larger surveys can be run, either with greatly improved response rates or superior methods are found for addressing the likely biases due to low response rates – challenges that are not unique to Natsal but that apply across the survey industry.

Results are exquisitely sensitive to assumptions about specificity of the LCx assay. More work could possibly be done synthesising results from earlier literature. This is a difficult area: most assessments of test accuracy in the absence of a ‘gold standard’ use a ‘composite reference standard’ [27, 28], a method that is known to always produce biased results [29].

On the issue of non-participation biases, the Natsal surveys, especially when viewed in conjunction with other sources of information on CT epidemiology may themselves be a wealth of information on the mechanisms of non-response, and on the risk factors for CT infection, even though as this paper suggests, estimates of absolute prevalence may be of very limited use. For example, changes in reported sexual behaviour between Natsal-2 and -3, in which number of partners, and extent of risky practices increased, although not to a great extent [30], would lead us to expect a rise in CT prevalence. Another example is that although 55% of 16–24 years old women reported being tested for CT during the previous year [31], the reported coverage of testing in the NCSP was only 41% [32], which includes what may be up to 31% repeat tests [33]. Thus, relative to the general population, a far higher proportion of Natsal-3 responders would have been tested and if CT positive they would have been treated, lowering the CT prevalence in responders, and hence the observed CT prevalence quite considerably.

These and other biases could be potentially addressed by selection modelling [34]. Heckman-type selection models although uncommon in the biomedical literature have been used for more than three decades in economics and social science literature [35]. The approach relies on a correlation parameter linking survey participation and infection status. Model identifiability improves by including selection variables obeying an exclusion restriction, to the effect that variables that are strong predictors of survey participation but are unrelated to infection status.

An alternative approach to monitoring CT prevalence in the UK has been proposed [36, 37], which relies on a three-compartment model of infection and diagnosis, and which makes use of the UK data on coverage of testing and diagnosis rates. The results are not incompatible with Natsal findings. However, the core assumption which allows changes in coverage and diagnosis rates to be mapped into changes in prevalence is that treatment-seeking and diagnosis-seeking behaviour has stayed constant. Whether this is indeed the case is debatable [38] but would seem unlikely as increased knowledge about the risk of chlamydia and its consequences as a result of the local and national awareness campaigns linked to the NCSP is likely to have increased diagnostic testing-seeking behaviour in asymptomatic women after a potential exposure [39]. In addition, the results point to little change in prevalence after 2000, until 2008–2010, which coincides with the date when the methods for recording coverage and diagnosis rates changed [32]. However, now that comprehensive recording systems are routinely providing consistent routine data, this method could be further refined, especially if it could be modified to incorporate data on health-seeking behaviour.

Another alternative might be to develop serological markers [12] of previous or recent infection in carefully chosen sentinel populations, such as women attending GU Medicine clinics or antenatal care. These are not general population samples, but they are samples which do suffer from non-response bias, in relatively stable populations, whose sexual and reproductive health are especially relevant. Time trends in age-specific markers of CT infection in such populations would be highly informative for policy makers and could be measured with precision and accuracy.

## Supplementary material

For supplementary material accompanying this paper visit https://doi.org/10.1017/S0950268819000347. click here to view supplementary material
