# Supplementary material for: Has Chlamydia trachomatis prevalence in young women in England, Scotland and Wales changed? Evidence from national probability surveys
Source: Epidemiol Infect. 2019 Mar 4;147:e107. doi: 10.1017/S0950268819000347 (PMC6518515; doi:10.1017/S0950268819000347)
Supplement: Supplementary file 1 [file S0950268819000347sup001.docx]

**Appendix 1: Preliminary analysis of sensitivity and specificity of the LCx assay assumed by Woodhall (2015) (11)**

The WinBUGS code and data in Appendix 3 was used, with the following code substituted for sensitivity and specificity of the LCx assay used in 2000, using data from Gaydos(20). A burn-in of 20000 was used and inference is based on 200,000 samples from two chains.

# sensitivity and specificity influenced by prevalence

se[1]~ dbeta(72,7) # sensitivity of LCx 91.9%

fp[1] ~ dbeta(4,423) # false positive rate of LCx 99.1%

# sensitivity and specificity NOT influenced by prevalence

se0 ~ dbeta(72,7) # sensitivity of LCx 91.9%

fp0 ~ dbeta(4,423) # false positive rate of LCx 99.1%

se[1] <- cut(se0)

fp[1] <- cut(fp0)

}

The results (Table S1) show that adjustment for sensitivity and specificity as in previous work (11), lowers age-specific CT prevalence estimates by between 0.7% and 2.7%. If the CT prevalence is not allowed to impact on specificity, the data is clearly incompatible with the sensitivity and specificity information ($\bar{D}=14.6$). If prevalence is allowed to influence the posterior estimates of sensitivity and specificity, the effect is to slightly shift the false positive rate downwards, making poor specificity less plausible, but even then the data is not compatible with the adjusted specificity ($\bar{D}=9.1$). Sensitivity estimates are not affected.

| Table S1. Observed CT prevalence in Natsal-2 by age, adjusted for sensitivity and specificity, with Residual Deviance goodness of fit statistics^a^ | | | | | |
| --- | --- | --- | --- | --- | --- |
|  |  | Prevalence impacts on accuracy estimates | | Prevalence has no impact accuracy estimates | |
| Age group | Observed | Adjusted Prevalence | Deviance | Adjusted  Prevalence | Deviance |
| 18-19 | 3.9 | 1.6 | 1.9 | 2.0 | 2.8 |
| 20-24 | 4.3 | 3.1 | 1.5 | 3.5 | 1.7 |
| 25-29 | 2.8 | 1.1 | 1.9 | 1.3 | 4.2 |
| 30-34 | 1.9 | 0.3 | 1.1 | 0.4 | 3.9 |
| 35-44 | 0.7 | 0.0 | 2.8 | 0.0 | 2.0 |
| Total Deviance |  |  | 9.1 |  | 14.6 |
| Posterior Sensitivity |  | 91.1  (83.9 – 96.3) |  | 91.1  (84.0– 96.3) |  |
| Posterior Specificity |  | 98.7  (98.0 - 99.4) |  | 99.1  (97.9 – 99.7) |  |
| ^a^ The fit is considered good if the summed deviance approximates the number of data elements, in this case 5 | | | | | |

**Appendix 2: Sensitivity of the AC2 assay in Natsal-3**

The performance of the AC2 assay in urine has been studied in previously (20,22-24). The published sensitivity estimates range from 93.6 to 100. We carried out a random effects synthesis using WinBUGS 1.4.3, with the following code and data

model{

for (i in 1:5) {

r[i] ~ dbin(p[i],n[i])

p[i] ~ dbeta(a,b) }

a ~ dexp(.001)

b ~ dexp(.001)

mu <- b / (a + b) }

}

list(a=10, b=100)

list(a=20, b=50)

r[] n[]

0 75 # Gaydos 2004

2 80 # Gaydos 2013

11 208 # Gaydos 2003

4 115 # Taylor 2011

54 850 # Gaydos 2010

END

Convergence occurred within 20,000 iterations based on Gelman-Rubin statistics available in WinBUGS. We discarded the first 40,000 iterations, and based posterior summaries on two chains with 100,000 samples.

The posterior distribution of sensitivity based on two chains with 100,000 samples each, had a mean 0.9495 (95%CrI 0.934 -0.965)

**Appendix 3: WinBUGS code for main analyses and for sensitivity analyses**

model {

for (i in 1:11) {

lpi[1,i] ~ dnorm(-3,.01)

logit(pi[1,i]) <- lpi[1,i]

r[i] ~ dbin(pi[1,i],n[i]) # likelihood pi[1,i]

lpi[2,i] ~ dnorm(-3,.01)

r2[i] <- r[i]

logit(pi[3,i]) <- lpi[2,i]

r2[i] ~ dbin(pi[2,i],n[i]) # likelihood pi[2,i]

rh[i] <- pi[2,i] * n[i] # expected numerator after Se/Fp adjustment

dev[i] <- (2 * (r[i] * (log(r[i]) - log(rh[i])) + (n[i]-r[i]) * (log(n[i]-r[i]) - log(n[i]-rh[i])))) # deviance

pi[2,i] <- pi[3,i] * se[J[i]] + (1-pi[3,i]) * fp[J[i]] # adjustment for sensitivity & false positive rate

pi[4,i] <- pi[3,i] * pse[i] # multiply by pr(sexual experience)

pi[5,i] <- pi[4,i] * rw[i]/nw[i] # balancing weights

pi[6,i] <- pi[4,i] * resp[J[i]] + (1-resp[J[i]]) * pi[7,i] # non-response adjustment

logit(pi[7,i]) <- logit(pi[4,i]) + lor[J[i]] # CT prev in non-responders

}

se11 <- se21 - 0.015

se12 <- se21 - 0.030

se21 ~ dnorm(.9495,15767) # sensitivity AC2 94.95 % from meta-analysis

fp12 ~ dbeta(1,1)

fp21 ~ dbeta(1,1)

se[1] <- se11 #  **BASECASE**

se[2] <- se21 # **BASECASE**

fp[1] <- .001 + fp12 * 0.002 # specifiicty LCx U(99.7 - 99.9)  **BASECASE**

fp[2] <- fp21 * .002 # specificty AC2 U(99.8 - 100)% **BASECASE**

lor[1] ~ dnorm(0.39, 29.4) # ClaSS CT prev LOR non-responders : responders **BASECASE**

lor[2] <- lor[1] # Natsal-3 non-response adjustment same as -3 **BASECASE**

resp[1] <- 0.42 # overall response rate Natsal-2

resp[2] <- 0.34 # overall response rate Natsal-3

# fp[1] <- .001 + fp12 * 0.008 # specificity LCX U(99.1 - 99.9) **SA 1**

# se[1] <- se12 # sensitivity LCx 3% lower than AC2  **SA 2**

#lor[1] ~ dnorm(0.642, 29.4) # Higher non-response correction **SA 3, SA 4**

**#** lor[2] <- lor[1] + log(1.1) # Natsal-3 OR 10% higher than Natsal-2 **SA4, SA5**

dev[12] <- sum(dev[1:5])

dev[13] <- sum(dev[6:11])

# summaries

for (k in 1:6) { av[1,k] <- (2*pi[k,1] + 5*pi[k,2]) / 7 # average 18-24 Natsal 2

av[2,k] <- (2*pi[k,7] + 5*pi[k,8]) / 7 # average 18-24 Natsal 3

av[3,k] <- (av[2,k] - av[1,k]) # average 18-24 Natsal 3 - Natsal 2 difference

av[4,k] <- av[3,k] * 100 / av[1,k] # average 18-24 % change from Natsal 2

av[5,k] <- (5*pi[k,3]+5*pi[k,4] +10*pi[k,5]) / 20 # average 25-44 Natsal 2

av[6,k] <- (5*pi[k,9]+5*pi[k,10] +10*pi[k,11]) / 20 # average 25-44 Natsal-3

av[7,k] <- (av[6,k] - av[5,k]) # average 25-44 Natsal 3 - Natsal 2 difference

av[8,k] <- av[6,k] * 100 / av[5,k] } # average 25-44 % change from Natsal 2

}

}

# INITIAL VALUES

list( fp12=.7, fp21=.7, se21=.8, lor=c(0,NA),

lpi=structure(.Data=c(-2,-2,-2,-2,-2, -2,-2,-2,-2,-2, -2,

-2,-2,-2,-2,-2, -2,-2,-2,-2,-2, -2),.Dim=c(2,11)) )

list( fp12=.3, fp21=.2, se21=.3, lor=c(1,NA),

lpi=structure(.Data=c(-4,-4,-4,-4,-4, -4,-4,-4,-4,-4, -4,

-4,-4,-4,-4,-4, -4,-4,-4,-4,-4, -4),.Dim=c(2,11)) )

list(J=c(1,1,1,1,1, 2,2,2,2,2,2))

r[] n[] pse[] rw[] nw[]

4 103 0.96 1.03792375 1.106935922 #Natsal 2 18-19

12 276 0.98 0.62289375 1.013115217 # 20-24

11 400 0.99 0.595229636 0.775895 # 25-29

9 472 1 0.593021556 0.781242161 # 30-34

6 804 1 0.6918125 0.85495 # 35-44

5 171 1 0.392 0.492345029 #Natsal 3 16-17

11 224 0.81 0.551090909 0.578370536 # 18-19

21 597 0.93 0.494 0.642247906 # 20-24

16 650 0.98 0.572 0.634084615 # 25-29

7 496 0.99 0.480285714 0.800350806 # 30-34

2 527 1 1.177 1.666332068 # 35-44

END
